# Evaluation of an initiative to improve advance care planning for a home-based primary care service

**DOI:** 10.1186/s12877-021-02035-x

**Published:** 2021-02-02

**Authors:** Michelle B. Cox, Margaret J. McGregor, Madison Huggins, Paige Moorhouse, Laurie Mallery, Katie Bauder

**Affiliations:** 1 Department of Family Practice, University of British Columbia, 713-828 West 10th Avenue, Vancouver, BC V5Z 1M9 Canada; 2 HomeViVE Program, Vancouver General Hospital, Vancouver, BC Canada; 3 Division of Geriatric Medicine, Dalhousie University, Halifax, NS Canada

**Keywords:** Advance care planning, Home-based primary care, Frailty, Substitute decision-maker, Do-not-resuscitate, Do-not-hospitalize

## Abstract

**Background:**

Advance care planning (ACP) is a process that enables individuals to describe, in advance, the kind of health care they would want in the future. There is evidence that ACP reduces hospital-based interventions, especially at the end of life. ACP for frail older adults is especially important as this population is more likely to use hospital services but less likely to benefit from resource intensive care. Our study goal was to evaluate whether an approach to ACP developed for frail older adults, known as the Palliative and Therapeutic Harmonization or PATH, demonstrated an improvement in ACP.

**Methods:**

The PATH approach was adapted to a primary care service for homebound older adults in Vancouver, Canada. This retrospective chart review collected surrogate measures related to ACP from 200 randomly selected patients enrolled in the service at baseline (prior to June 22, 2017), and 114 consecutive patients admitted to the program after implementation of the PATH ACP initiative (October 1, 2017 to May 1, 2018). We compared the following surrogate markers of ACP before and after implementation of the PATH model, chart documentation of: frailty stage, substitute decision-maker, resuscitation decision, and hospitalization decision. A composite ACP documentation score that ascribed one point for each of the above four measures (range 0 to 4) was also compared. For those with documented resuscitation and hospitalization decisions, the study examined patient/ substitute decision-maker expressed preferences for do-not-resuscitate and do-not-hospitalize, before and after implementation.

**Results:**

We found the following changes in ACP-related documentation before and after implementation: frailty stage (27.0% versus 74.6%, *p* < .0001); substitute decision-maker (63.5% versus 71.9%, *p* = 0.128); resuscitation decision documented (79.5% versus 67.5%, *p* = 0.018); and hospitalization decision documented (61.5% versus 100.0%, *p* < .0001); mean (standard deviation) composite ACP documentation score (2.32 (1.16) versus 3.14 (1.11), *p* < .0001). The adjusted odds ratios (95% confidence intervals) for an expressed preference of do-not-resuscitate and do-not-hospitalize after implementation were 0.87 (0.35, 2.15) and 3.14 (1.78, 5.55), respectively.

**Conclusions:**

Results suggest partial success in implementing the PATH approach to ACP in home-based primary care. Key contextual enablers and barriers are important considerations for successful implementation.

## Background

Advance care planning (ACP) refers to “the process of discussing and recording preferences concerning goals of care for patients who may lose capacity or communication ability in the future” [1]. ACP is associated with improved symptom management [2], increased caregiver satisfaction [3, 4], and reduced hospital use at the end of life [5]. Nevertheless, studies show that providers lack the confidence and competency to initiate ACP conversations [6], especially for frail populations [7]. Advance care preferences arise from the ACP process. These include an expressed preference in the event of an acute health crisis, for inclusion or avoidance of: life-extending procedures such as cardio-pulmonary resuscitation and intubation; transfer to hospital; home-based therapeutic interventions; and/or comfort care and palliation. We call these preferences rather than directives because the ultimate decision for a preferred intervention rests with the most responsible provider based on weighing the medical appropriateness in various circumstances.

Frailty is a “multidimensional syndrome of loss of reserves (e.g., energy, physical, ability, cognition, health) that gives rise to vulnerability” (p. 489 [8]). Frailty, more than age, predicts poor surgical outcomes [9], longer hospital stays, institutionalization [10], and death [10]. Failure to adequately consider these outcomes with frailty during ACP has the potential to favour life prolonging measures over symptom control and quality of life [11]. Despite these important considerations, providers report a disproportionate hesitancy to engage in ACP with frail older patients compared to those with cancer [12].

The Palliative and Therapeutic Harmonization (PATH) model was developed by two geriatricians in Nova Scotia, Canada [13] to increase engagement in ACP with frail older adult populations and their families in inpatient and outpatient settings. This study aims to evaluate the implementation of a PATH approach to ACP in a home-based primary care program for frail homebound older adults in Vancouver, Canada.

Interest for implementing the PATH model originated amongst primary care clinicians, nurses, and allied health providers working in a home-based primary care program run by the Vancouver Coastal Health Authority. Primary care providers identified the need for improved ACP after attending presentations on the PATH approach. They noted its high relevance to their practice population, which focuses on the care of frail older adults. The staff sought and successfully obtained donor funding to support the training and implementation of a PATH approach to ACP.

### Clinical context

Home Visits for Vancouver’s Elders (HomeViVE) is a home-based primary care program for frail older adults who are unable to access usual primary care as a result of dementia and/or physical frailty. Patients are followed longitudinally by a ‘most responsible provider’ (e.g., family physician or nurse practitioner working with the HomeViVE program), supported by a team of registered nurses and allied health providers (physiotherapists and occupational therapists) as well as office administrative support.

Services include regularly scheduled routine home visits, responsive day-time and after-hours care for emergencies, nursing assistance, physical and occupational rehabilitative services, and palliative support. The service caseload includes approximately 400 patients. Within the patient group, there is a monthly average of four transfers to long-term care and seven deaths, two thirds of which occur in the home.

A majority of patients in the HomeViVE program also receive home care support, case management, and other services from the publicly run community home care system. Community home care providers (registered nurses, physiotherapists, occupational therapists) record their clinical notes in an electronic medical record (EMR) that is outside the HomeViVE primary care system. This means that notes written by community nurses, physiotherapists, and occupational therapists are not seen by attending HomeViVE primary care providers. Likewise, when a HomeViVE physician or nurse practitioner visits a patient, the community providers are not able to read their EMR visit notes.

### Institutional and health system context

Physicians working in the HomeViVE home-based care program are remunerated through a publicly-funded fee-for-service model run by the provincial government medical services payment plan. Nurse practitioners, nurses, allied health staff and administrative staff are salaried employees, hired by the regional health authority (Vancouver Coastal Health). The after-hours on-call component of the service is provided by the physician group who receive a stipend for this service supplemented by the fee-for-service remuneration.

Patients identified as needing HomeViVE services are referred by the community home care system operated through the public health authority. Criteria for admission include residence in Vancouver, age over 80 years, home-bound status, inability to access usual primary care due to physical or cognitive frailty, and the presence of a high degree of frailty based on dependency on others for basic activities of daily living. Patients who enrol in the service agree to seek all primary care from the HomeViVE physician or nurse practitioner assigned to them through the service. While some HomeViVE patients are directly case-managed by the HomeViVE nurses, a majority are case-managed by the community home care system. Home care support is not part of the HomeViVE service and is delivered through the community home care system.

Based in a large urban Canadian setting, over one third of HomeViVE patients do not speak English as their first language. The program has tried to address language barriers through the use of translated written ACP materials, and the frequent deployment of publicly funded 24/7 phone-based and (less frequently) in-person interpretation services.

The HomeViVE program was chosen as the setting in which to implement a PATH ACP process because it had a cohesive team that provided care to a frail population, capacity to provide around-the-clock primary care, and a high degree of interest among the physician group to implement a PATH approach.

## Methods

### Intervention

PATH is an innovative model designed to improve the patient/family experience and resource utilization in frail people. The model enables health care teams to understand and respond to frailty, and empowers patients and families with information that helps them navigate the complexity of frailty, including the health crisis. Table 1 presents a summary of the PATH approach.

**Table 1:** Palliative and Therapeutic Harmonization (PATH) Approach

**UNDERSTAND**	**Understand health status** through comprehensive geriatric assessment, including cognition (stage of dementia), mobility, function, nutrition, social situation, medical illnesses, medications, and trajectory.
**COMMUNICATE**	**Exchange of information.** Provide detailed information to patients and/or family. Describe each illness and its trajectory; dementia and its stages; frailty stage; and risks associated with frailty.
**EMPOWER**	**Empower patient and/or family** to make informed decisions, including what to do during a health crisis (i.e., an acute decline in health status). Engage patient and/or family-member in directed decision making.
**RESPOND**	**Respond** to the health crisis, when it occurs, applying knowledge of frailty stage and advance care plans.

The PATH ACP approach is based on evidence that frailty, more than age, is one of the strongest predictors of health outcomes [10]. Training for the PATH ACP intervention involved the completion of two online training modules, followed by a 2 day in-person training session. The modules introduced learners to the concept of frailty, the importance of understanding frailty domains (e.g., cognition, mobility, and function), and how to stage frailty to clarify prognosis and inform decision-making. Through case vignettes, participants became familiar with the concept of frailty, its importance as a driver of health outcomes in older adults, and the staging of frailty (mild, moderate, severe and very severe) based on the Clinical Frailty Scale [8]. The 2 day in-person training, delivered by the PATH founders, reinforced and built upon these concepts by engaging learners in the hands-on practice of Collaborative Comprehensive Geriatric Assessment (CoCGA) with volunteer patients (and their informal caregiver to provide collateral), recruited from the HomeViVE program.

The training emphasized the importance of identifying a substitute decision-maker (SDM), an individual(s) who makes health care decisions on behalf of the patient should the patient lack the capacity to make decisions on their own during an acute health crisis (i.e., sudden worsening of health), or by virtue of dementia. Identification of an SDM is crucial when care providers respond to a health crisis after hours, so they can communicate with this person if needed. Beyond an SDM, it is also important to identify people within the patient’s informal “circle of care” who may participate in the informal decision-making process or might be affected by the decisions made. The combination of the patient, and their identified formal and informal decision-making “circle of care” will henceforth be referred to as the patient/SDM(s) dyad.

An important element of the PATH model is a commitment to communicating the results of the holistic health assessment and providing a realistic explanation of prognosis to empower the patient, or their SDM where appropriate, to make informed current and future health decisions. The communication strategy aims to educate the SDM and those in the patient’s circle of care about (1) the concept of frailty, (2) cognitive status, (3) the patient’s current frailty stage, (4) the prognosis associated with frailty, (5) the potential for and anticipated types of health crises, and (6) the risks and benefits of interventions and standard treatments when patients are frail. These communication skills were practiced using simulation with team debriefing and a PATH semi-structured conversation guide. Further details of the training content have been described elsewhere [13].

Following formal training, the second phase of the PATH ACP implementation (October 1, 2017 to May 1, 2018) involved 7 months of follow-up phone meetings between PATH trainers and the project implementation working group to discuss progress and challenges. Additionally, over the same period, during regular monthly meetings of the HomeViVE team, there was an agenda item to discuss clinicians’ experience and share stories of PATH ACP. Finally, a practice improvement coach, who was a registered nurse with considerable clinical and administrative experience in the care of older adults, reviewed the charts of new patients and reminded clinicians, through direct messaging in the EMR, when key ACP data elements (described below) were absent.

**Fig. 1:**
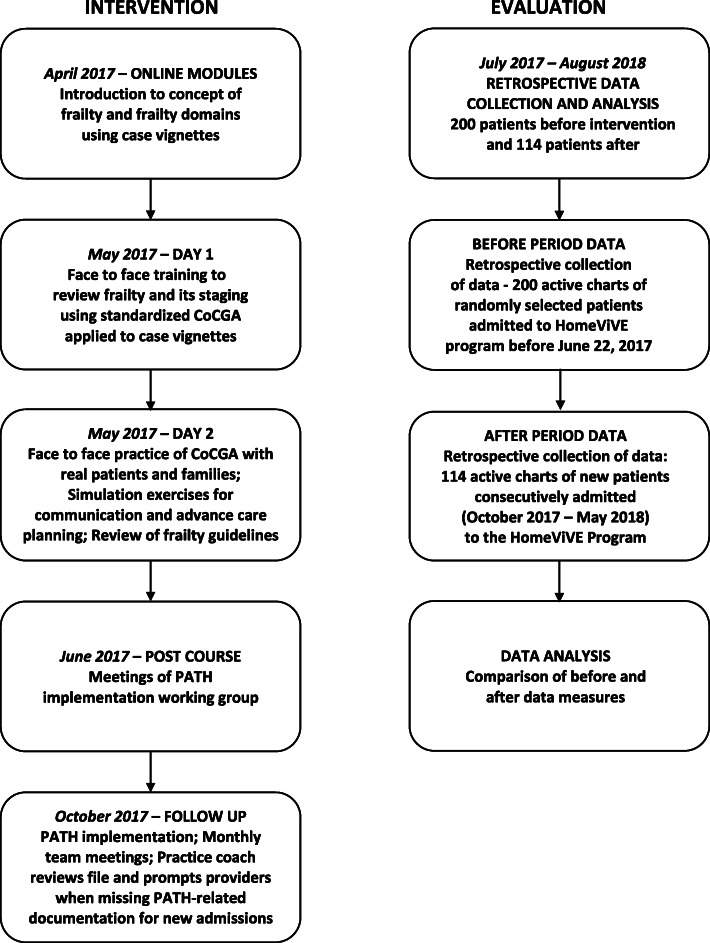
Palliative and Therapeutic Harmonization (PATH) Advance Care Planning Adapted to HomeViVE — Intervention and Evaluation Timelines. *CoCGA* – Collaborative Comprehensive Geriatric Assessment; *PATH* – Palliative and Therapeutic Harmonization; *HomeViVE* – Home Visits for Vancouver’s Elders home-based primary care program

Further practice tools were created to facilitate PATH ACP implementation, including (1) cards with a list of “key questions to ask during a health crisis”, printed in English and simplified Chinese, for families and patients to use, (2) e-forms for the PATH CoCGA, and (3) a decision-making guide formatted for the shared EMR. There was a time lag between the formal training period and when implementation began due to the summer break when team meetings were suspended, and many staff were away. Deployment of the above described tools was launched on October 1, 2017, 5 months after the formal training had ended. Figure 1 describes the PATH intervention and timelines.

### Data source and data measures

Evaluation of the PATH ACP intervention was based on retrospective chart review for the before implementation and after implementation periods. The before period used 200 randomly selected clinical EMRs of patients admitted to the HomeViVE program prior to June 22, 2017, which were gathered for a previous study [14]. A detailed description of this and analysis of ACP data at baseline has been previously published [14].

Data was abstracted, by a researcher, from the charts of 114 consecutive admissions, for newly enrolled patients in the HomeViVE program, between October 1, 2017 and May 1, 2018. All members of the HomeViVE primary care team were trained in the PATH approach so that every patient enrolled in the post implementation period benefitted from a PATH-trained primary care physician or nurse practitioner.

There was episodic review of data by a second team member to ensure inter-rater reliability. Consecutive admissions were used for the comparator group because, over the 7 month accrual period, there was an insufficient number of new admissions to adequately power a random selection process. Further, although clinicians likely began to implement the PATH ACP approach on existing patients, the team mutually agreed to begin PATH ACP on all newly admitted patients in a more systematic way on October 1, 2017 when the full team was present. Data collected after implementation was compared to the ACP baseline data. A description of the evaluation process and timeline is briefly summarized in Fig. 1.

The following documentation data variables were collected before and after the ACP intervention: (1) frailty stage documented; (2) name of SDM documented; (3) resuscitation decision documented; and (4) hospitalization decision documented. Documentation was deemed to be present if the key element was located at the front of the HomeViVE EMR or “face sheet”—a location mutually agreed upon by the provider team at the time of the PATH implementation. A composite documentation score was created by assigning one point to each of the above measures, with scores ranging from 0 (no measures documented) to 4 (all measures documented). The study hypothesis was that if there was a systematic approach to ACP conversations, more data reflecting this would be recorded on patients’ charts after implementation compared to before implementation.

In addition to documenting variables, data were also collected on the content of decision-making. This study examined the proportion of patient/ SDM(s) dyads who expressed a preference for do-not-resuscitate (DNR) and do-not-hospitalize (DNH), before and after implementation of the PATH approach. A second study hypothesis was that given the overall prevalence of moderate to severe frailty in the patient population, a standardized approach to frailty staging and communication may result in a greater proportion of expressed preferences to avoid resuscitation (DNR) and hospital interventions (DNH) in favour of a home-based approach to care. Table 2 describes all measured elements of PATH training implemented for this study.

**Table 2:** Measured Metrics of Palliative and Therapeutic Harmonization (PATH) Advance Care Planning Implementation

**Documentation of key element on EMR**	**Description**
Frailty stage documented^a^	Documentation on EMR of assigned frailty stage using the Clinical Frailty Scale (8) informed by Collaborative Comprehensive Geriatric Assessment.
Substitute decision-maker documented^a^	Documentation on EMR of the patient’s substitute decision-maker^b^ or formal representative, if available.
Resuscitation decision documented^a^	Documentation on EMR of patient decision with regards to resuscitation in the event of a cardiopulmonary arrest.
Hospitalization decision documented^a^	Documentation on EMR of patient decision with regards to hospitalization in the event of a health crisis.
**Expressed preferences**	**Description**
Expressed preference for do-not-resuscitate	The resuscitation decision expresses a preference for do-not-resuscitate (versus resuscitate).
Expressed preference for do-not-hospitalize	The hospitalization decision expresses a preference for do-not-hospitalize (versus hospitalize) in the event of an acute care crisis.

*EMR* electronic medical record

^a^Documented – refers to the presence of documentation on key advance care planning decisions; these preferences are noted on the EMR face sheet, which is the front page of the EMR that contains crucial patient information such as patient identification and personal information, clinical information, and identification of family/substitute decision-maker

^b^The substitute decision-maker is the person responsible to make health decisions on the patient’s behalf when there is impaired capacity. This person is designated in the absence of a formal representation agreement

Further data was collected on patient demographics (age, sex and main language spoken), co-morbidities and frailty stage (mild, moderate, severe, very severe) based on the Clinical Frailty Scale [8]. Main language spoken was identified based on a field in the EMR that recorded the main language used by the patient to communicate. Comorbidities were identified if they were listed in the problem list or medical history, and if not mentioned, were assumed to be absent. Missing data was explicitly measured for dementia as this diagnosis is often missed, especially when present in its milder form.

All data were de-identified and ethics approval was obtained from the University of British Columbia Behavioural Research Ethics Review Board and the Vancouver Coastal Health Research Institute ethics review board.

### Data analysis

This observational before/after study presents descriptive statistics on demographic and health characteristics of the study population. The analysis included unpaired comparative analyses before and after implementation of four measures of ACP documentation, one composite ACP documentation measure, and two measures of expressed ACP preference. These tests included the two independent samples t-test for continuous data, and the Chi-square test or Fisher’s exact test for categorical data.

Logistic regression analyses were conducted to explore the association between the post-implementation period and expressed preferences for DNR or DNH. Potential confounders were identified using univariate analyses and were entered into the models. Factors that remained significant (*p* < .05) in the multiple regression analysis were retained in the final models and odds ratios for the post-implementation period, adjusted for confounders, were determined.

Statistical analyses were conducted using SAS software, version 9.4 (SAS Institute Inc., Cary, NC, USA).

## Results

A total of eight physicians and three nurse practitioners participated in the full PATH training. Data were abstracted from 314 charts, 200 before implementation and 114 following implementation. The mean (standard deviation) age of patients was 88.1 (± 7.2) years. A little over one third (35.4%) were male and for a similar proportion (109/314, 34.7%) English was not their first language. Approximately 50.3% of patients had documented dementia and 19.4% were severely or very severely frail (Table 3).

**Table 3:** Patient Demographics and Comorbidities for HomeViVE Home-Based Primary Care Patient Cohorts (*N* = 314)

Patient demographics	Before^**a**^ ***N*** = 200	After^**a**^ ***N*** = 114	Total ***N*** = 314
Mean age in years (SD)	87.7 (7.1)	88.9 (7.2)	88.1 (7.2)
Minimum^b^ - maximum	60–106	69–106	60–106
Male, N (%)	65 (32.5%)	46 (40.4%)	111 (35.4%)
Main language spoken^c^, N (%)			
English	128 (64.0%)	73 (64.0%)	201 (64.0%)
Non-English^d^	69 (34.5%)	40 (35.1%)	109 (34.7%)
*Missing (*i.e.*, spoken language not identified)*	3 (1.5%)	1 (0.9%)	4 (1.3%)
**Patient comorbidities**			
Dementia diagnosis, N (%)			
Yes	103 (51.5%)	55 (48.3%)	158 (50.3%)
No	60 (30.0%)	11 (9.7%)	71 (22.6%)
*Missing (*i.e.*, no comment on the possibility of having dementia)*	37 (18.5%)	48 (42.1%)	85 (27.1%)
Chronic conditions, N (%)			
Arthritis	90 (45.0%)	36 (31.6%)	126 (40.1%)
Osteoporosis	63 (31.5%)	18 (15.8%)	81 (25.8%)
Congestive Heart Failure	51 (25.5%)	21 (18.4%)	72 (22.9%)
Stroke	50 (25.0%)	19 (16.7%)	69 (22.0%)
Frailty staging, N (%)			
Mild	3 (1.5%)	3 (2.6%)	6 (1.9%)
Moderate	24 (12.0%)	48 (42.1%)	72 (22.9%)
Severe	25 (12.5%)	30 (26.3%)	55 (17.5%)
Very Severe	2 (1.0%)	4 (3.5%)	6 (1.9%)
*Missing (*i.e.*, no frailty stage indicated in chart)*	146 (73.0%)	29 (25.4%)	175 (55.7%)

*HomeViVE* Home Visits for Vancouver’s Elders home-based primary care program, *SD* standard deviation

^a^Before period: Active patients enrolled in program prior to June 20, 2017; After period: New consecutive patients enrolled between October 1, 2017 and May 1, 2018

^b^Despite the age criteria for admission to the program being age over 80 years, there are occasional exceptions where this is waived

^c^Main language was identified based on the primary language used by the patient to communicate

^d^Non-English main languages spoken include: Cantonese, Czech, Finnish, German, Greek, Gujarati, Hindi, Italian, Punjabi, Italian, Mandarin, Persian, Polish, Portuguese, Serbian, Spanish, Tagalog, and Vietnamese

EMR documentation of frailty stage was 27.0% before implementation compared to 74.6% after (*p* < .0001). There was an increase in documentation of a substitution decision-maker(s) (63.5 to 71.9%, *p* = 0.128) that was not statistically significant. Likewise, there was increased documentation about whether to pursue hospital-based care (61.5 to 100%, *p* < .0001). In contrast, following the intervention, documentation of resuscitation code status decreased from 79.5 to 67.5% (*p* = 0.018). The mean (standard deviation) composite documentation score (i.e., the sum of the four measures, ranging from 0 to 4), was 2.32 (1.16) before versus 3.14 (1.11) after the intervention, *p* < .0001 (Table 4).

**Table 4:** Presence of Advance Care Planning Documentation Among HomeViVE Home-Based Primary Care Patients, Before (*N* = 200) and After (*N* = 114) Palliative and Therapeutic Harmonization (PATH) Implementation

Documentation of key element on EMR	Before^**a**^ ***N*** = 200	After^**a**^ ***N*** = 114	***p***-value^**b**^
Frailty stage documented^c^, N (%)	54 (27.0%)	85 (74.6%)	**<.0001**
Substitute decision-maker documented^c^, N (%)	127 (63.5%)	82 (71.9%)	0.128
Resuscitation decision documented^c^, N (%)	159 (79.5%)	77 (67.5%)	**0.018**
Hospitalization decision documented^c^, N (%)	123 (61.5%)	114 (100.0%)	**<.0001**
Mean (SD) composite documentation score^d^	2.32 (1.16)	3.14 (1.11)	**<.0001**

*HomeViVE* Home Visits for Vancouver’s Elders home-based primary care program, *EMR* electronic medical record, *SD* standard deviation

^a^Before period: Active patients enrolled in program prior to June 20, 2017; After period: New consecutive patients enrolled between October 1, 2017 and May 1, 2018

^b^Tests of comparison included: two independent samples t-test for continuous data; Chi-square test or Fisher’s exact test for categorical data

^c^Documented – refers to the presence of documentation on key advance care planning decisions; these preferences are noted on the EMR face sheet, which is the front page of the EMR that contains crucial patient information such as patient identification and personal information, clinical information, and identification of family/substitute decision-maker

^d^Composite documentation score – measure of overall documentation; each documented measure (frailty stage, substitute decision maker, resuscitation decision, hospitalization decision) is assigned one point if documented in the patient record; the composite score is the sum of all measures; score ranges from 0 to 4

Expressed preference for DNR was not statistically different before and after implementation of the PATH process (90.6% versus 88.3%, *p* = 0.591), whereas expressed preference for DNH increased (23.6% before and 51.8% following the initiative, *p* < 0.0001) (data not shown). Separate multiple regression models were run for an expressed DNR preference and an expressed DNH preference. There was no significant effect on expressed preference for DNR after the intervention, and age and osteoporosis remained significant in the final model. There was a significant effect on expressed preference for DNH after the intervention even after adjusting for age, male gender, and relevant comorbidities (adjusted odds ratio 3.14 (95% confidence interval 1.78, 5.55)) (Table 5).

**Table 5:** Logistic Regression, Adjusted Odds Ratios for Factors Associated with Expressed Preferences Among HomeViVE Home-Based Primary Care Patients (*N* = 236)

**Do-not-resuscitate preference**	**Adjusted OR (95% CI)**^**a**^
After period^b^	0.87 (0.35, 2.15)
Age	**1.09 (1.02, 1.17)**
Male	0.91 (0.36, 2.33)
Osteoporosis	**4.61 (1.02, 20.92)**
**Do-not-hospitalize preference**	**Adjusted OR (95% CI)**^**a**^
After period^b^	**3.14 (1.78, 5.55)**
Age	**1.04 (1.00, 1.09)**
Male	1.44 (0.79, 2.63)
Arthritis	**0.50 (0.27, 0.90)**

*HomeViVE* – Home Visits for Vancouver’s Elders home-based primary care program;* OR* – odds ratio; *CI* – confidence interval

^a^Other variables tested for significance in univariate logistic regression analyses for DNR preference included: English is main language spoken; dementia diagnosis; arthritis; congestive heart failure; stroke; advanced frailty. Other variables tested for significance for DNH preference included: English as main language spoken; dementia diagnosis; osteoporosis; congestive heart failure; stroke; advanced frailty. Stepwise multiple logistic regression models included any variables that were significant in univariate logistic regression analysis (*p* < .05) but were removed from model if they did not remain significant. Final models were adjusted for age and sex regardless of significance

^b^After period variable is a binary variable that records whether patient is enrolled in before period (active patients enrolled in program prior to June 20, 2017) or the after period (new consecutive patients enrolled in program between October 1, 2017 and May 1, 2018)

## Discussion

This study evaluated the implementation of a PATH approach to ACP in a home-based primary care program for frail older adults. It measured the proportion of patients with chart documentation of key surrogate measures of ACP and expressed decision-making preferences for DNR and DNH, before and after the intervention.

Compared to before the PATH intervention, there was significant improvement in the EMR documentation of frailty stage, and documentation of a hospitalization decision in the after period. Although not statistically significant, the proportion of patients having a documented SDM listed on their EMR increased from 63.5 to 71.9%. While EMR documentation of these variables does not necessarily reflect the quality of ACP communication, the variables are reasonable surrogate indicators of ACP, and their increase following PATH training suggests more proactive ACP occurred as a result of the training.

One exception to the above findings is the observed paradoxical decline in the documentation of a resuscitation decision in the post-implementation period compared to baseline. This finding may relate to the relative focus that PATH communication places on educating families and patients regarding hospital interventions rather than cardio-pulmonary resuscitation, which is a relatively rare event even when individuals are frail and homebound. This may also reflect a presumed DNR for patients who opted for a DNH. In addition, documentation of a resuscitation decision was high at baseline (79.5%) and was recorded in over two thirds of cases post implementation, despite the observed decline.

This analysis demonstrated a significant increase in the proportion of patient/ SDM(s) dyads expressing a preference to avoid hospital (DNH) in favour of home-based care interventions for future health crises, which increased from 23.6% before to 51.8% after implementation. Although the actual rate of hospital transfer before and after implementation was not measured, patients’ recorded preferences to avoid cardiopulmonary resuscitation and hospitalization have been shown to result in lower rates of hospital admission [1], an outcome that is desirable in frail populations if symptoms can be managed in community settings.

Beyond the empirical results, some lessons about the importance of context and institutional support for practice change were learned through informal discussions with participating clinicians, course instructors, program administrators and practice coaches over the course of the project, and from a qualitative evaluation report on ACP discussions at HomeViVE [15]. Context enablers of the initiative included the enthusiasm of the provider team and access to on-call after-hours care. The ongoing support and advice of the PATH geriatricians during the implementation period, and the deployment of a practice coach who provided clinicians with feedback data on their ACP metrics in real-time were additional enablers.

A contextual barrier to PATH ACP implementation in the home-based primary care setting was the fee-for-service remuneration method for family physicians. Family physicians, who comprised the majority of the primary care providers, and fee-for-service remuneration of these practitioners likely discourages comprehensive geriatric assessment and ACP communication, which require extensive time commitments. Another identified barrier was the requirement that the PATH model, as it was originally conceived, delegate a substantial portion of the assessment for staging frailty to trained allied health providers. Despite an administrative agreement to implement the PATH ACP initiative in the home-based primary care program, reassigning nursing or allied heath staff to this role was not supported by the program administrator, leaving the burden of ACP activities to the most responsible primary care physician or nurse practitioner. The PATH ACP approach, therefore, had to be carried out longitudinally using shorter, cumulative assessments by the most responsible primary care provider, instead of having other members of the team complete the CoCGA in more extended visits. Additional systemic barriers include the dual charting system, for home-based clients, between the HomeViVE and home care team, and lack of inter-operability between these systems.

As with all studies there were some limitations. First, data was not extracted from a reference population of patients over the same time period. It is, therefore, possible that the observed changes may be a result of other ACP interventions, unrelated to the PATH ACP initiative, taking place within the health region at that time. In addition, other key elements of the PATH ACP approach, such as the use of the PATH form that guides decision-making about future health crises and documentation of preferred place of death, were not measured. This study did not measure hospital utilization, the patient and family experience, clinical satisfaction with PATH intervention, or the cost-effectiveness and sustainability of the program. All of these measures could be important areas for future research to evaluate ACP practice improvement interventions.

Despite these limitations, this study had a number of strengths including the fact that it was implemented in the community, whereas most published research evaluating ACP initiatives take place in nursing home or hospital settings [1]. A second strength is the sample size, which was relatively large compared to other evaluative studies of ACP interventions [1]. Furthermore, in a systematic review [16] of ACP evaluation studies, none of the studies discussed implementation challenges nor explored how the intervention may have differed in practice compared to what was originally envisaged. As such, the description of contextual elements discussed here present new insights. Finally, the results and findings of this study are generalizable to other longitudinal home-based primary care programs for frail older adults operating in Canada [17, 18] and the US [19, 20].

## Conclusion

In conclusion, evaluation of the PATH ACP initiative suggests a promising approach to ACP in the setting of home-based primary care of frail older adult populations. As with all complex adaptive systems, further implementation and scaling of the approach needs to consider key contextual enablers and barriers.

## Data Availability

The datasets used and analyzed during the current study are available from the corresponding author upon reasonable request.

## Abbreviations

ACP: Advance care planning
CI: Confidence interval
CoCGA: Collaborative Comprehensive Geriatric Assessment
DNR: Do-not-resuscitate
DNH: Do-not-hospitalize
EMR: Electronic medical record
HomeViVE: Home Visits for Vancouver’s Elders
OR: Odds ratio
PATH: Palliative and Therapeutic Harmonization
SD: Standard deviation
SDM: Substitute decision-maker
